# Important radiological and clinicopathological risk factors for the recurrence of intraductal papillary mucinous neoplasms after surgical resection

**DOI:** 10.1007/s00330-025-11431-5

**Published:** 2025-02-19

**Authors:** Junghoan Park, Jung Hoon Kim, Rae Rim Ryu, Sungjun Hwang

**Affiliations:** 1 Department of Radiology, Seoul National University Hospital, Seoul, Republic of Korea; 2 Department of Radiology, Seoul National University College of Medicine, Seoul, Republic of Korea; 3 Institute of Radiation Medicine, Seoul National University Medical Research Center, Seoul, Republic of Korea; 4 Department of Radiology, Chung-Ang University Hospital, Seoul, Republic of Korea; 5 Department of Radiology, Inje University Ilsan Paik Hospital, Goyang, Republic of Korea

**Keywords:** Pancreas, Pancreatic intraductal neoplasm, Tomography (X-ray computed), Recurrence, Proportional hazards models

## Abstract

**Objectives:**

To assess significant radiological and clinicopathological risk factors for post-surgery recurrence in patients with intraductal papillary mucinous neoplasm (IPMN).

**Materials and methods:**

Patients with IPMNs who underwent surgery from 2011 to 2021 at a single center were retrospectively included. Two reviewers evaluated CT findings according to international guidelines. Clinicopathological data were collected from medical records and surgical pathology reports. Patients were monitored for recurrence with contrast-enhanced CT or MRI up to 2023. Univariable Cox regression analysis included potential risk factors: all high-risk stigmata and worrisome features in the international guidelines, age, sex, tumor location, type, carcinoembryonic antigen, surgery type, postsurgical residual cyst, adjuvant treatment, pathologic grade, type, size, margin status, lymph node metastasis, gland type, and pancreatic intraepithelial neoplasia. Variables with *p* < 0.2 were included in multivariate analysis.

**Results:**

Among 332 patients (mean age, 66.3 ± 9.0 years; 212 men), recurrence occurred in 39 (11.7%) over a median follow-up of 3.2 years (range: 0.1–12.3 years). Two- and five-year recurrence-free survival rates were 91.2% and 86.4%, respectively. Significant radiological risk factors included enhancing mural nodule (EMN) presence (hazard ratio [HR] 5.088, *p* = 0.007) and lymphadenopathy (HR 2.837, *p* = 0.01). Associated invasive carcinoma (HR 25.030), lymph node metastasis (HR 27.562), adjuvant treatment (HR 0.203), and history of pancreatitis (HR 2.608) were also significant. Most imaging features showed moderate to excellent interobserver agreement, except for thickened/enhancing cyst walls (κ, 0.25).

**Conclusion:**

The presence of EMNs and lymphadenopathy, along with several clinicopathologic factors, were significantly associated with IPMN recurrence.

**Key Points:**

***Question*** *Understanding postoperative recurrence risk in IPMN patients is crucial for determining surveillance strategies; however, research on radiologic risk factors remains limited*.

***Findings*** *The presence of EMNs and lymphadenopathy were identified as significant radiologic risk factors for the postoperative recurrence of IPMN, along with clinicopathologic factors*.

***Clinical relevance*** *IPMN recurrence is significantly associated with imaging findings like EMNs and lymphadenopathy, as well as clinical and pathologic factors. It can guide the development of tailored postoperative surveillance strategies for IPMN patients in further studies*.

**Graphical Abstract:**

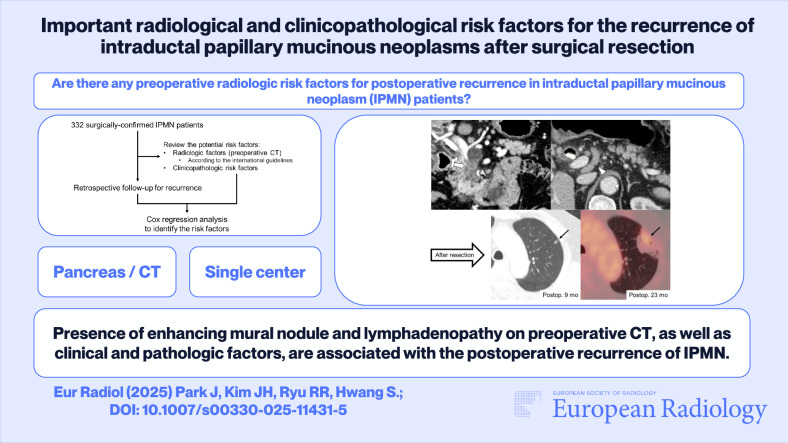

## Introduction

The detection frequency of cystic lesions in the pancreas has increased with advancement and the widespread use of cross-sectional imaging, with many lesions thought to be intraductal papillary mucinous neoplasms (IPMNs) [1]. Although the majority of IPMNs are benign, some have malignant potential, making it crucial to establish management and surveillance strategies for these lesions. As a result, several studies have addressed this issue, leading to the development and revision of international consensus guidelines (ICGs) for IPMNs [1, 2]. The ICGs define high-risk stigmata and worrisome features to aid in surgical decision-making for IPMN patients [1, 2]. However, determining surveillance strategies for patients after surgical resection remains a challenge.

Currently, it is known that even noninvasive IPMN can lead to the development of clinically significant remnant pancreatic lesions (such as pancreatic ductal adenocarcinoma or IPMN which are indications for resection) after partial pancreatectomy, with risks persisting even beyond 5 years post-surgery [3–5]. Consequently, recent guidelines recommend lifetime surveillance for all patients who have undergone surgery for IPMN, including those diagnosed with IPMN with low-grade dysplasia (LGD), as long as they remain fit for surgical resection [2, 6]. However, these recommendations are not strongly supported by high-level evidence, such as randomized controlled trials. Although rare, some studies suggest a low recurrence rate in noninvasive branch-duct IPMNs, indicating that surveillance might be discontinued for selected patients [7]. Therefore, further research on risk-based surveillance strategies is needed, and understanding the factors that influence recurrence is crucial. Several studies have investigated risk factors for postoperative recurrence of IPMN, suggesting multiple potential factors, including pathologic grade, family history of pancreatic cancer, and other clinicopathologic factors [3, 4, 7–11]. However, despite the importance of cross-sectional imaging in the diagnosis and management of IPMNs, few studies have thoroughly evaluated preoperative radiologic features alongside clinical and pathological factors as potential risk factors for postoperative recurrence. Even when some radiologic features are included in the analysis, the results vary among studies, highlighting the need for further research on the impact of preoperative imaging features on postoperative recurrence [4, 10, 11].

Therefore, this study aims to identify risk factors affecting postoperative recurrence-free survival in IPMN patients through a survival analysis that includes preoperative imaging findings, as well as pre- and postoperative clinical and pathological features.

## Materials and methods

This study was approved by the institutional review board of our hospital, and informed consent was waived due to its retrospective nature.

### Patients

**Fig. 1:**
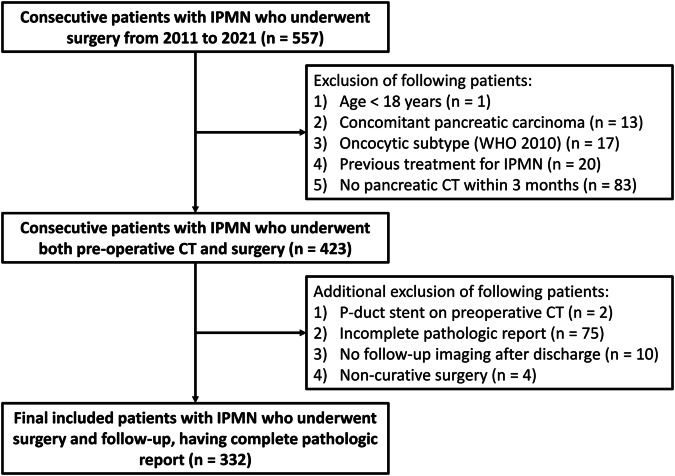
The diagram for patient inclusion. IPMN, intraductal papillary mucinous neoplasm

We retrospectively identified 557 consecutive patients who were histologically diagnosed with IPMN after surgical resection at our hospital from January 2011 to September 2021, which constitutes the same group of patients as in our previous study [12]. Initially, we excluded 134 patients based on the following criteria consistent with our previous study [12]: under 18 years of age (*n* = 1), presence of concomitant focal solid or cystic pancreatic lesions other than IPMN (*n* = 13), diagnosis of the oncocytic subtype of IPMN (*n* = 17), history of receiving treatment for IPMN (*n* = 20), and absence of preoperative CT within 3 months before surgery (*n* = 83). We further excluded 91 patients based on the following additional criteria: (1) the presence of a pancreatic duct stent on preoperative CT (*n* = 2); (2) the absence of any key information in the postoperative pathology report, including pathologic grade, gross type, pathologic size, margin status, lymph node metastasis, gland type, and the presence of pancreatic intraepithelial neoplasia (PanIN) (*n* = 75); (3) the absence of follow-up abdominal CT or MRI after discharge (*n* = 10); or (4) noncurative surgery (i.e., the presence of a gross residual invasive lesion or metastasis at the time of surgery) (*n* = 4). Patients in whom only benign cysts without high-risk stigmata or worrisome features remained after surgery were considered as having undergone curative surgery and were not excluded. Finally, a total of 332 patients were included, of whom 234 were patients from our previous study [12], and 98 were not included (Fig. 1).

### Image acquisition and analysis

All patients underwent preoperative pancreatic CT via multidetector CT scanners. At our hospital, pancreatic CT consists of three or four phases: precontrast, early arterial (optional), late arterial, and portal venous phases. Each patient received 1.5 mL/kg nonionic contrast material at a rate of 3–5 mL/s via an automatic power injector. A bolus-tracking method was used for image acquisition, with a region of interest placed at the descending aorta and a trigger threshold set at 100 Hounsfield units. The average scanning delays were 23 s for the early arterial phase, 37–45 s for the late arterial phase, and 70 s for the portal venous phase. All CT scans included at least one contrast-enhanced coronal or oblique coronal reconstruction image and might also include sagittal reconstruction and curved multiplanar reconstruction images following the pancreatic duct. Pancreatic CTs from other hospitals were accepted if they included at least three phases (precontrast, late arterial phase, and portal venous phase) and at least one coronal or oblique coronal reconstruction image among the postcontrast images. Since our study was retrospective, the scanners and acquisition parameters used varied, as summarized in the Supplementary Material.

**Fig. 2:**
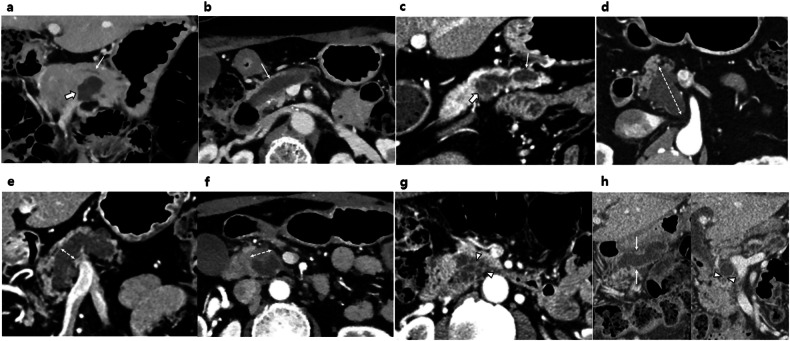
Representative images illustrating radiologic features of IPMN. **a** Branch-duct type IPMN with a gross cyst (thick arrow) but without MPD dilatation (arrow). **b** Main-duct type IPMN with dilated MPD (arrow) without gross cyst. **c** Combined type IPMN with both a gross cyst (thick arrow) and MPD dilatation (arrow). **d** Cyst size, **e** MPD diameter, and **f** EMN size measured at the longest diameter in either axial, coronal, or oblique coronal images (dashed arrows). **g** Thickened/enhancing cyst wall (arrowheads), and **h** abrupt caliber change of pancreatic duct (arrowheads) with distal pancreatic atrophy (arrows)

Two board-certified abdominal radiologists (J.H.K. and S.H., with 20 years and 7 years of experience in abdominal radiology, respectively) independently reviewed the preoperative pancreatic CT images of all patients. All CT images, including those from other hospitals, were extracted as DICOM files from the Picture Archiving Communication System of our institution for the reviewers. The reviewers used RadiAnt DICOM Viewer (Medixant) software to load the DICOM files and review the CT images. The following imaging features were analyzed: gross type, cyst size, main pancreatic duct (MPD) diameter, presence and size of enhancing mural nodules (EMNs), presence of thickened/enhancing cyst walls, presence of abrupt caliber changes in the pancreatic duct with distal pancreatic atrophy, and presence of lymphadenopathy (Fig. 2). The results from these independent reviews were used to evaluate interobserver agreement. Then, a consensus was reached between the reviewers for the final decision in cases of significant discrepancies in measurements or any discrepancies in binary features. This consensus-based final decision was subsequently used for risk factor evaluation. Both reviewers were aware that these patients were diagnosed with IPMN, but they were blinded to other clinical and pathologic information, including recurrence status and pathologic grade. Definitions for each feature are summarized in Supplementary Table 1.

### Clinical and pathological data collection

Clinical data were obtained through electronic medical records and preoperative and immediate postoperative imaging studies. The following information was collected: age at surgery, sex, tumor location, presence of obstructive jaundice, history of pancreatitis, serum levels of carcinoembryonic antigen and carbohydrate antigen 19-9, type of surgery, presence of gross residual cysts after surgery, and receipt of adjuvant treatment (chemotherapy and/or radiation therapy). Obstructive jaundice was defined as a tumor located in the pancreas head with a total serum bilirubin level greater than 1.5 mg/dL or the presence of a biliary stent. Gross residual cysts after surgery were defined as those remaining 5 mm or larger on immediate postoperative imaging. The pathological data included the pathological grade, gross type, pathological size, margin status, presence of lymph node metastasis, gland type, and presence of PanIN, as mentioned above. These data were obtained from postoperative pathology reports written by board-certified pathologists.

### Follow-up and definition of recurrence

After curative resection, each patient followed a standardized follow-up protocol, which involved contrast-enhanced CT or MRI every 6–12 months, along with monitoring of carbohydrate antigen 19-9 level to assess tumor recurrence. However, the intervals varied based on the clinical judgment of the attending physician and the patient’s condition. We accepted contrast-enhanced abdominal CT and enhanced or nonenhanced pancreatobiliary MRI with or without chest CT as valid surveillance modalities. Follow-up was conducted until November 2023 or until recurrence occurred. Recurrence was defined as one or more of the following: (1) the development of new IPMNs or the progression of existing IPMNs in the remnant pancreas requiring surgical intervention, including cases where surgery was performed or high-risk stigmata occurred but surgery was not performed due to patient factors; (2) the occurrence of pancreatic ductal adenocarcinoma in the remnant pancreas; and (3) the occurrence of metastasis in locations other than the pancreas. Both radiological and histological diagnoses were included, but cases with only indeterminate lesions on imaging were not considered recurrence unless confirmed as such by follow-up imaging or tissue examination. The time of recurrence was determined from the point at which it was first suspected in the radiologic report.

### Statistical analysis

Univariable Cox regression analysis was conducted to identify potential risk factors for recurrence after surgery for IPMN. The analysis included all clinical, radiologic, and pathologic factors. Factors with *p* < 0.2 in the univariable analysis were then included in a multivariable Cox regression analysis to identify independent risk factors for postoperative recurrence of IPMN, with a significance level set at *p* < 0.05. The proportional hazards assumption was verified using the Schoenfeld method, and a step function was employed in cases where the proportional hazards assumption was violated [13]. For radiologic factors, interobserver agreement between two reviewers was evaluated based on the results of their independent reviews prior to reaching a consensus, using either Cohen’s kappa (for qualitative variables) or the intraclass correlation coefficient (ICC, for continuous variables). The Cohen’s kappa was categorized as poor (< 0.0), slight (0.0–0.2), fair (0.2–0.4), moderate (0.4–0.6), substantial (0.6–0.8), or almost perfect (> 0.8), while the ICCs were categorized as poor (< 0.5), moderate (0.5–0.75), good (0.75–0.9), or excellent (> 0.9) [14, 15]. All the statistical analyses were performed using R statistical software (version 4.2.0; R Foundation for Statistical Computing).

## Results

### Patient characteristics

The mean age of the 332 patients included in the study was 66.3 ± 9.0 years, and 120 were female. Approximately 60% of the patients had tumors in the pancreatic head, and the majority of these patients (56% of the total patients) underwent pylorus-preserving pancreatoduodenectomy. Thirty-one percent underwent distal pancreatectomy, while approximately 8% underwent total pancreatectomy. The interval between preoperative CT and surgery averaged 16.0 ± 21.1 days.

The mean follow-up period after surgery was 3.54 ± 2.36 years (range, 0.10–12.32 years), during which 39 patients (12%) experienced recurrence. Among these patients, 8 had intrapancreatic recurrence, while the remaining 31 had extrapancreatic recurrence: five had local recurrence, nine had liver metastasis, seven had lung metastasis, four had peritoneal seeding, three had lymph node metastasis, one had pleural seeding, and two had multiple regions affected. The 2-year and 5-year recurrence-free survival rates were 91.2% and 86.4%, respectively (Table 1).

**Table 1:** Baseline characteristics of the subjects

Variables	Number or mean ± SD
Total subjects	332
Age at operation (years)	66.3 ± 9.0
Sex	
Male	212 (63.9%)
Female	120 (36.1%)
Tumor location	
Head	199 (59.9%)
Body or tail	116 (34.9%)
Diffuse	17 (5.1%)
Obstructive jaundice	8 (2.4%)
History of pancreatitis	39 (11.7%)
CEA (ng/mL)	6.7 ± 81.2
CEA > 5 ng/mL	15 (4.5%)
CA 19-9 (U/mL)	50.4 ± 233.1
CA 19-9 > 37 U/mL	54 (16.3%)
Surgical procedure	
Whipple’s operation	11 (3.3%)
PPPD	185 (55.7%)
Duodenum-preserving pancreatic head resection	1 (0.3%)
Distal pancreatectomy	104 (31.3%)
Central pancreatectomy	6 (1.8%)
Total pancreatectomy	25 (7.5%)
Gross residual cyst after surgery	61 (18.4%)
Adjuvant treatment	60 (18.1%)
Days from CT to surgery	16.0 ± 21.1
Recurrence	39 (11.7%)
Intrapancreatic	8 (2.4%)
Extrapancreatic	31 (9.3%)
Follow-up duration (years)	3.54 ± 2.36

The numbers in parentheses represent the ratio to the total number of subjects

*SD* standard deviation, *CEA* carcinoembryonic antigen, *CA 19-9* carbohydrate antigen 19-9, *PPPD* pylorus-preserving pancreaticoduodenectomy

### Radiologic and pathologic features of IPMN

Preoperative CT scans revealed that over half of the patients had combined-type IPMN, with an average cyst size ranging from 31.8–32.1 mm, depending on the reviewer, and an average MPD diameter ranging from 7.3–7.4 mm. Slightly fewer than half (44–47%) had EMNs, with an average EMN size of 15.8 mm. Lymphadenopathy was uncommon (4–7%). Overall, 177 patients (53.3%) had at least one high-risk stigmata in a consensus: 145 had EMNs ≥ 5 mm, 92 had MPD diameters ≥ 10 mm, and 8 had obstructive jaundice. Among the patients without high-risk stigmata, 136 (41.0%) had worrisome features, whereas 19 (5.7%) did not have any high-risk stigmata or worrisome features according to the 2017 ICGs. The interobserver agreement between the two readers was mostly moderate to excellent (ICC, 0.77–0.91; κ, 0.49–0.69), except for the assessment of thickened/enhancing cyst walls, which showed fair agreement (κ, 0.25) (Table 2).

**Table 2:** Radiologic features of the IPMNs

Variables	Radiologist 1	Radiologist 2	Agreement (ICC^a^ or kappa^b^)
Gross type			0.69 (0.62–0.77)^b^
Branch-duct	102 (30.7%)	119 (35.8%)	
Main-duct	24 (7.2%)	19 (5.7%)	
Combined	206 (62.0%)	194 (58.4%)	
Cyst size (mm)^†^	31.8 ± 16.0	32.1 ± 15.7	0.81 (0.78–0.85)^a^
MPD diameter (mm)^†^	7.4 ± 5.4	7.3 ± 5.7	0.91 (0.89–0.93)^a^
EMN presence	147 (44.3%)	156 (47.0%)	0.61 (0.52–0.69)^b^
EMN size (mm)^†^	7.0 ± 10.3	7.4 ± 10.4	0.77 (0.72–0.81)^a^
Excluding IPMNs without EMN	15.8 ± 10.0	15.8 ± 9.9	
Thickened/enhancing cyst wall	117 (35.2%)	176 (53.0%)	0.25 (0.15–0.35)^b^
Abrupt change in caliber of the pancreatic duct with distal pancreatic atrophy	46 (13.9%)	60 (18.1%)	0.51 (0.38–0.63)^b^
Lymphadenopathy	23 (6.9%)	12 (3.6%)	0.49 (0.28–0.70)^b^

*EMN* enhancing mural nodule, *ICC* intraclass correlation coefficient, *MPD* main pancreatic duct

^a^ ICC with a 95% confidence interval in parentheses

^b^ Cohen’s kappa with a 95% confidence interval in parentheses

^†^ Data are presented as mean ± standard deviation. Otherwise, data are presented as numbers (ratio to the total number of subjects)

Postoperative pathological examination revealed that 15% of patients had IPMNs with high-grade dysplasia (HGD) and 27% had associated invasive carcinoma. IPMNs with LGD were found in 58% of patients. Most patients had negative resection margins; among the 58 patients (17%) with positive margins, most had LGD involvement, while only 2 patients had HGD, and 7 had invasive carcinoma. Among these, recurrence occurred in 10 patients; 8 of them had an extrapancreatic recurrence, and 1 had an intrapancreatic recurrence but developed at a site distant from the resection margin, which was considered a true recurrence. The remaining patient had soft tissue development at the resection margin; however, there was no soft tissue present immediately after surgery, and the diagnosis was made 1700 days later, indicating a high likelihood of being a true recurrence. Lymph node metastasis was found in 28 patients (8%). The gland type was gastric in 63% of the patients, with the remainder being intestinal, pancreatobiliary, mixed, or undetermined. PanIN was present in 166 patients (50%), mostly low-grade PanIN, with high-grade PanIN in only 16 patients (5%) (Table 3).

**Table 3:** Pathologic features of the IPMNs

Variables	Number or mean ± SD
Pathologic grade	
LGD	193 (58.1%)
HGD	50 (15.1%)
Associated invasive carcinoma	89 (26.8%)
Gross type	
Branch-duct	117 (35.2%)
Main-duct	29 (8.7%)
Combined	186 (56.0%)
Pathologic size (mm)	40.6 ± 28.1
Margin status	
Negative	274 (82.5%)
LGD	49 (14.8%)
HGD	2 (0.6%)
Invasive carcinoma	7 (2.1%)
Lymph node metastasis	28 (8.4%)
Gland type	
Gastric	208 (62.7%)
Intestinal	50 (15.1%)
Pancreatobiliary	12 (3.6%)
Gastric + intestinal	31 (9.3%)
Gastric + pancreatobiliary	25 (7.5%)
Intestinal + pancreatobiliary	4 (1.2%)
Gastric + intestinal + pancreatobiliary	1 (0.3%)
Undetermined	1 (0.3%)
PanIN	
None	166 (50.0%)
Low-grade	150 (45.2%)
High-grade	16 (4.8%)

The numbers in parentheses represent the ratio to the total number of subjects

*PanIN* pancreatic intraepithelial neoplasia, *SD* standard deviation

### Important radiological and clinicopathological risk factors for the recurrence of IPMN after surgery

Table 4 summarizes the univariable and multivariable Cox regression analyses for recurrence-free survival after surgery for IPMN. According to the univariable analysis, patient age was not significantly associated with recurrence risk (hazard ratio [HR], 1.011; *p* = 0.54), whereas sex showed borderline significance (HR, 1.630 for female sex; *p* = 0.13). Among the other clinico-laboratory features, obstructive jaundice (HR, 4.808; *p* = 0.009), history of pancreatitis (HR, 2.188; *p* = 0.049), and adjuvant treatment (HR, 9.196; *p* < 0.001) were potentially significant risk factors. Among the radiologic features, MPD diameter, the presence and size of EMN, abrupt change in caliber of the pancreatic duct with distal pancreatic atrophy, and lymphadenopathy were confirmed as potentially significant risk factors (*p* < 0.05), whereas gross type showed borderline significance (*p* = 0.19). Among the pathologic features, HGD (HR, 14.798; *p* = 0.02) and associated invasive carcinoma (HR, 90.991; *p* < 0.001) presented a significantly greater risk than IPMN with LGD. Margin status, nongastric gland type, and PanIN status were also identified as potentially significant risk factors (*p* < 0.05). Lymph node metastasis did not satisfy the proportional hazards assumption (*p* = 0.03), necessitating the division of the analysis based on a 2-year postoperative period, as indicated by the Schoenfeld residual plot (Supplementary Fig. 1). Within 2 years after surgery, LN metastasis was potentially significant (HR, 40.943; *p* < 0.001), whereas beyond 2 years, it was borderline significant (HR, 3.493; *p* = 0.13). Gross type and pathologic size also showed borderline significance (*p* < 0.2).

**Table 4:** Univariable and multivariable Cox regression analysis for recurrence-free survival after surgery for IPMN

Variables	Univariable analysis			Multivariable analysis		
	HR	95% CI	*p*-value	HR	95% CI	*p*-value
Clinico-laboratory features						
Age at operation (years)	1.011	0.976–1.047	0.54			
Female sex	1.630	0.868–3.062	0.13			
Tumor location			0.41			
Head	1.000		Ref.			
Body or tail	1.462	0.757–2.824	0.26			
Diffuse	1.873	0.545–6.441	0.32			
Obstructive jaundice	4.808	1.473–15.702	0.009			
History of pancreatitis	2.188	1.002–4.778	0.049	2.608	1.123–6.053	0.03
Log (CEA) (ng/mL)	2.726	1.561–4.759	< 0.001			
Log (CA 19-9) (U/mL)	3.002	1.968–4.577	< 0.001			
Total pancreatectomy	1.248	0.429–3.627	0.69			
Gross residual cyst after surgery	1.176	0.356–2.035	0.72			
Adjuvant treatment	9.196	4.781–17.687	< 0.001			
Before 2 years				0.203	0.064–0.648	0.007
After 2 years				1.947	0.469–8.094	0.36
Radiologic features						
Gross type (radiologic)			0.19			
Branch-duct	1.000		Ref.			
Main-duct	2.196	0.639–7.551	0.21			
Combined	2.138	0.933–4.898	0.07			
Cyst size (mm)	1.001	0.981–1.021	0.93			
MPD diameter (mm)	1.054	1.010–1.099	0.02			
EMN presence	10.084	3.925–25.909	< 0.001	5.088	1.549–16.712	0.007
EMN size (mm)	1.054	1.035–1.072	< 0.001			
Thickened/enhancing cyst wall	0.881	0.444–1.749	0.72			
Abrupt change in caliber of the pancreatic duct with distal pancreatic atrophy	2.113	1.028–4.344	0.04			
Lymphadenopathy	4.723	2.221–10.042	< 0.001	2.837	1.279–6.295	0.01
Pathologic features						
Pathologic grade			< 0.001			
LGD	1.000		Ref.	1.000		Ref.
HGD	14.798	1.653–132.469	0.02	8.797	0.961–80.538	0.054
Associated invasive carcinoma	90.991	12.437–665.704	< 0.001	25.030	3.036–206.378	0.003
Gross type (pathologic)			0.07			
Branch-duct	1.000		Ref.			
Main-duct	3.275	1.209–8.872	0.02			
Combined	1.773	0.816–3.851	0.15			
Pathologic size (mm)	1.006	0.997–1.014	0.18			
Margin status			< 0.001			
Negative	1.000		Ref.			
LGD	0.857	0.331–2.218	0.75			
HGD	4.655	0.630–34.411	0.13			
Invasive carcinoma	10.856	3.749–31.430	< 0.001			
Lymph node metastasis						
Before 2 years	40.943	17.794–94.162	< 0.001	27.562	8.572–88.621	< 0.001
After 2 years	3.493	0.704–17.328	0.13	0.687	0.109–4.316	0.69
Non-gastric gland type	3.823	1.934–7.554	< 0.001			
PanIN			< 0.001			
None	1.000		Ref.			
Low-grade	0.556	0.260–1.189	0.13			
High-grade	4.888	2.199–10.865	< 0.001			

Radiologic features were based on analysis results by a consensus

*CA 19-9* carbohydrate antigen 19-9, *CEA* carcinoembryonic antigen, *CI* confidence interval, *EMN* enhancing mural nodule, *MPD* main pancreatic duct, *PanIN* pancreatic intraepithelial neoplasia

**Fig. 3:**
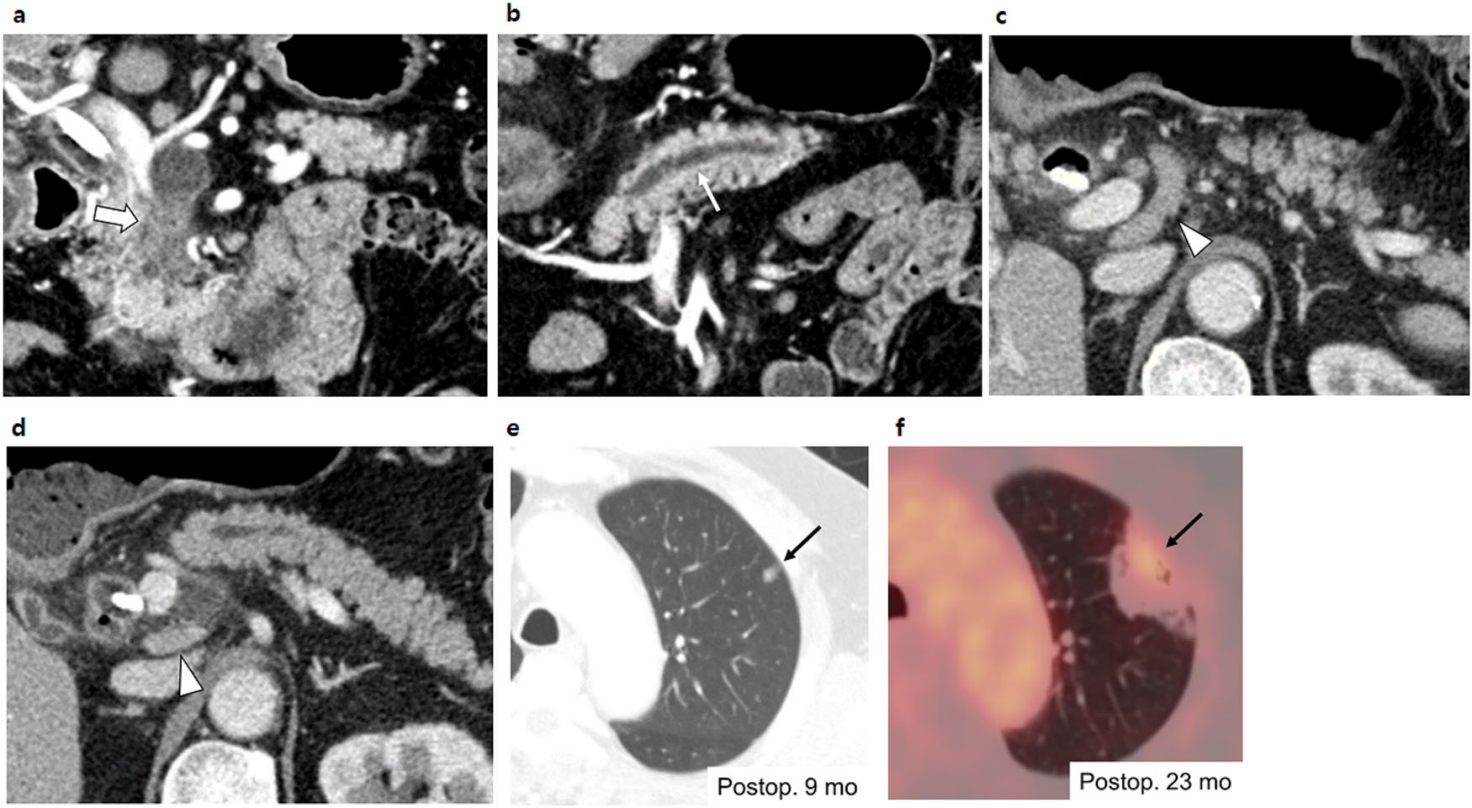
A representative case of a 57-year-old male patient who underwent surgery for IPMN**. a** On the preoperative CT, there was an approximately 5 cm pleomorphic cystic lesion with an EMN (thick arrow) in the uncinate process of the pancreas, which was thought to be IPMN with high-risk stigmata. **b** The MPD (white arrow) was mildly dilated to 5 mm, and **c, d** several enlarged lymph nodes (arrowheads) were observed around the lesion. After surgical resection, the lesion was confirmed to be IPMN with an associated invasive carcinoma, and two metastatic lymph nodes were identified. **e** During postoperative follow-up, a small lung nodule (black arrow) developed 9 months after surgery, which was reported to be a possible metastasis. **f** This was confirmed to be a metastasis as it had increased in size and F-18 fluorodeoxyglucose uptake (black arrow) on follow-up imaging

**Fig. 4:**
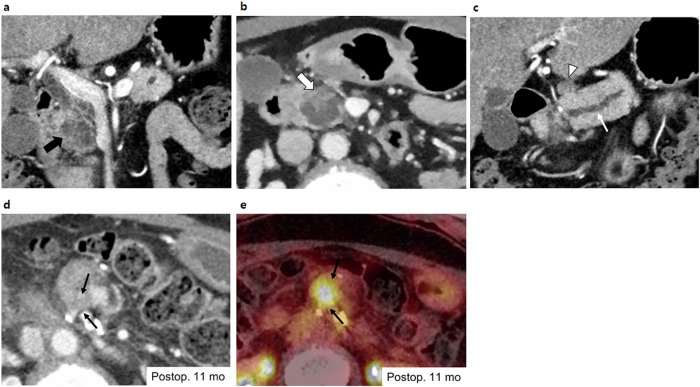
Another representative case of a 57-year-old female patient who underwent surgery for IPMN. **a** On the preoperative CT, there was a 3.2 cm pleomorphic cystic lesion in the pancreas head (thick black arrow). **b** The lesion had about 1.8 cm EMN (thick white arrow). **c** The MPD (white arrow) was mildly dilated to 6 mm. A prominent peripancreatic lymph node (arrowhead) was observed; however, as the short diameter was less than 10 mm, it was not considered lymphadenopathy. After surgical resection, the lesion was confirmed to be IPMN with an associated invasive carcinoma, and 11 metastatic lymph nodes were identified. **d** During postoperative follow-up, a hypoenhancing soft tissue lesion developed around the pancreaticojejunostomy site (black arrow) 11 months after surgery, which was reported to be a tumor recurrence. **e** This was confirmed by increased F-18 fluorodeoxyglucose uptake (black arrow) on PET/CT

**Fig. 5:**
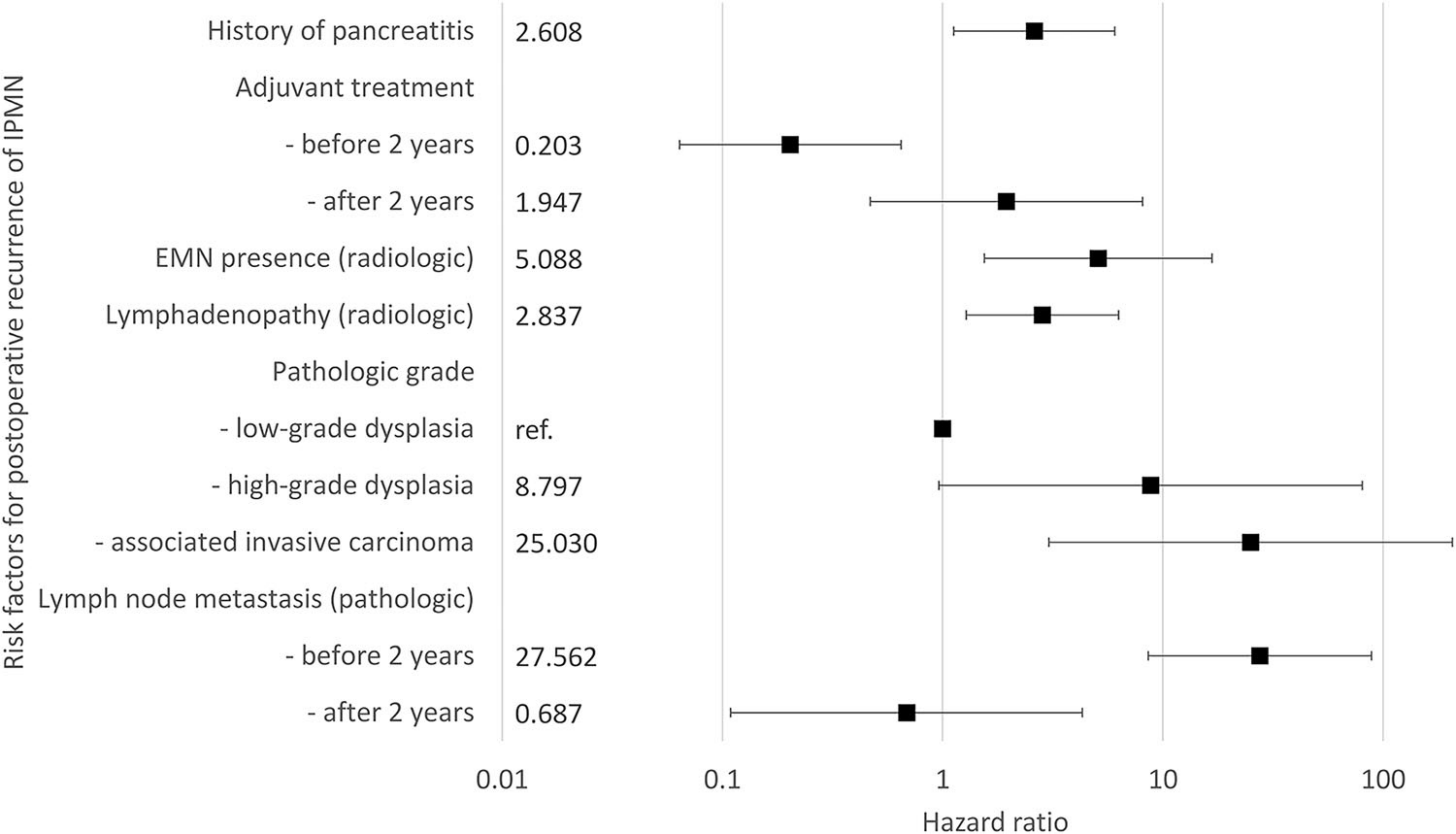
The forest plot for the independently significant risk factors for postoperative recurrence of IPMN. EMN, enhancing mural nodule

**Fig. 6:**
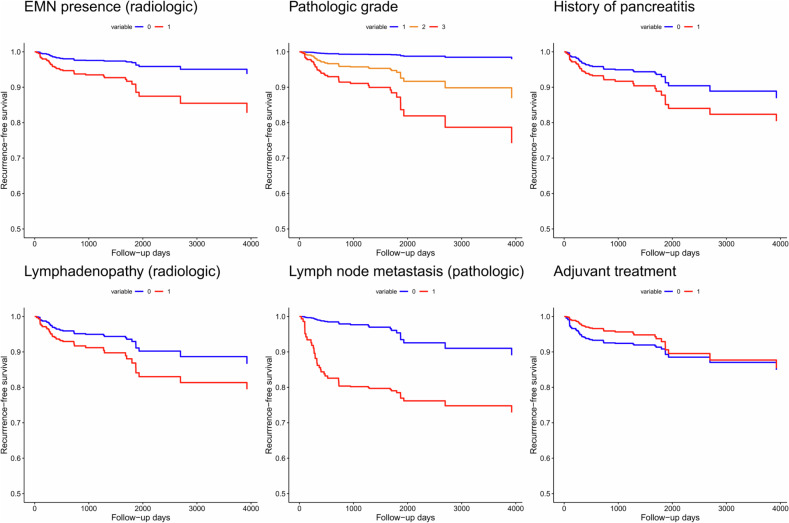
The adjusted Kaplan–Meier curves for the independently significant risk factors for postoperative recurrence of IPMN. For pathologic grade, blue, orange, and red indicate LGD, HGD, and associated invasive carcinoma, respectively. For the other features, blue indicates the absence of the feature while red indicated the presence of the feature. EMN, enhancing mural nodule

In the multivariate analysis, the presence of EMNs (HR, 5.088; *p* = 0.007) and lymphadenopathy (HR, 2.837; *p* = 0.01) were independently significant radiologic risk factors (Figs. 3 and 4). Additionally, associated invasive carcinoma (HR, 25.030; *p* = 0.003), lymph node metastasis (HR, 27.562; *p* < 0.001 within the 2-year postoperative period), history of pancreatitis (HR, 2.608; *p* = 0.03), and adjuvant treatment (HR, 0.203; *p* = 0.007 within the 2-year postoperative period) were found to be significant independent risk factors. Adjuvant treatment did not satisfy the proportional hazard assumption in the multivariable analysis (*p* = 0.002), so the analysis was divided based on the 2-year postoperative period. Both lymph node metastasis and adjuvant treatment were significant factors for predicting recurrence within 2 years after surgery but not thereafter (Table 4 and Fig. 5). The adjusted Kaplan‒Meier curve also revealed that adjuvant treatment was a negative risk factor for IPMN recurrence in the early postoperative period, whereas the presence of EMNs, lymphadenopathy, higher pathological grade, lymph node metastasis, and history of pancreatitis were positive risk factors (Fig. 6).

## Discussion

Our study revealed that specific preoperative imaging features, including the presence of EMN and lymphadenopathy, are significant risk factors for predicting postoperative recurrence of IPMN, alongside clinical and pathological features. Although IPMN with associated invasive carcinoma (HR, 25.030) and lymph node metastasis (HR, 27.562 within the 2-year postoperative period) appeared to be the most powerful risk factors, the presence of EMN and radiologic evidence of lymphadenopathy remained significant imaging-based risk factors even after adjusting for these pathologic features. The 5-year recurrence-free survival rate was 86.4%, which is slightly higher than that reported in previous studies (75–82%) [7, 10, 16, 17]. This difference is likely due to variations in surgical indications or the definition of recurrence.

Several studies have investigated factors influencing the postoperative recurrence of IPMN. Although various potential risk factors have been proposed, pathologic grade has been consistently identified as a significant risk factor [3, 4, 7–11]. Our study also revealed that invasive carcinoma was a strong risk factor. Although HGD did not reach statistical significance (HR, 8.797; *p* = 0.054), this may be due to the relatively small number of recurrence cases. A larger sample size could identify HGD as a significant factor.

In contrast, few studies have explored radiologic features as potential risk factors for IPMN recurrence. In our study, the presence of EMNs emerged as the most powerful imaging-based risk factor. While EMNs are well-known predictors of malignancy risk in IPMN, their impact on recurrence is less understood [2]. One study revealed that EMN size on preoperative MR images affects patient prognosis, but pathological features were not included in the multivariable analysis; thus, it is unclear whether EMN size is an independent prognostic factor, independent of pathological grade [18]. Two other studies examined the impact of mural nodules ≥ 5 mm on recurrence via endoscopic ultrasound; one study found them to be a significant risk factor in both univariate and multivariate analyses, while the other found them significant only in univariate analysis, with no significance in multivariate analysis [10, 11]. In contrast to these studies, our research utilized contrast-enhanced CT, a widely-used noninvasive imaging method for pancreas evaluation, and identified the significance of EMNs in multivariable analysis after adjusting for clinical and pathologic factors. This may further substantiate mural nodules as an independent predictor of postoperative recurrence. Nevertheless, while EMNs may be associated with gross malignant components which potentially impact prognosis, previous studies have revealed that a significant proportion of HGDs or the most severe dysplasia are detected outside of mural nodules via histologic examination [19, 20]. Therefore, further research is needed to explore the association and causal relationship between EMNs and IPMN recurrence.

With respect to lymph nodes, several studies have indicated that lymph node metastasis increases the risk of recurrence, which aligns with our findings [10, 17]. However, little attention has been given to whether lymphadenopathy observed on imaging independently increases the risk of recurrence. In our study, lymphadenopathy was determined based on size (short diameter ≥ 1 cm). Therefore, our finding that lymphadenopathy on imaging is an independent risk factor can be partially explained by the hypothesis that the size of metastatic lymph nodes influences prognosis. However, not all enlarged lymph nodes are metastatic [21]; further research through radiologic‒pathologic correlation is needed to determine whether enlarged lymph nodes increase the risk of recurrence regardless of metastasis.

Among the clinical features, history of pancreatitis was identified as a significant factor increasing the risk of recurrence. Previous studies have also reported that pancreatitis is associated with IPMN recurrence, and recent research has shown that the presence of symptoms is linked to an increase in both intra- and extrapancreatic recurrence, which is consistent with our findings [11, 22]. Finally, adjuvant treatment is known to improve survival in patients with invasive IPMN, especially those with metastatic lymph nodes [23–25]. In our study, it was also found to reduce early postoperative recurrence, which aligns with previous reports.

As mentioned, even noninvasive IPMNs can lead to the development of clinically significant remnant pancreatic lesions after surgical resection [2]. Some studies have even reported cases of extrapancreatic recurrence in patients initially diagnosed with noninvasive IPMN, including IPMN with LGD [10, 11, 16]. However, many patients do not experience recurrence (10-year disease-free survival, 70–78%), and a recent study reported a low recurrence rate for patients with IPMN with LGD (10-year recurrence rate, 3.7%) [7, 10, 17]. Therefore, predicting the risk of recurrence for individual IPMN patients and establishing an appropriate surveillance strategy are crucial. Our study results suggest that preoperative imaging not only aids in the differential diagnosis of cysts and the determination of surgical candidates but also has the potential to help predict postoperative prognosis and inform surveillance strategies. For example, extending the follow-up interval after surgical resection in patients without risk factors might be a practical approach, although further research is needed to validate risk factors across multiple institutions and to establish a specific follow-up strategy.

In this study, we used pancreatic CT for preoperative radiologic risk evaluation. While MRI plays a significant role in the diagnosis and management of IPMN, CT alone often provides sufficient evaluation, and a substantial number of patients at our institution proceed to surgery based solely on CT findings. To include more patients and reduce selection bias, we chose CT as our imaging modality, as it is also likely to support broader application compared to MRI. Our study has several limitations. First, it was a retrospective study conducted at a single center with a relatively small number of patients experiencing recurrence relative to the number of features analyzed. A large-scale multicenter study would be needed to enhance the validity of our findings. Second, pathologic features were obtained from pathology reports rather than by rereviewing specimens, which may introduce interobserver variability due to the involvement of various reporting pathologists. There is also a risk of selection bias, as a substantial number of patients with incomplete pathology reports were excluded. Additionally, patient inclusion and the definition of recurrence depend on surgical indications, which can vary between surgeons and institutions, potentially leading to variation in the results. Finally, the definition and timing of recurrence are not always clear because we rely on radiologic reports to determine recurrence. Indeterminate lesions on imaging may lead to ambiguity, and in some cases, recurrence may be detected later than its actual occurrence because it is missed during interpretation. However, such indeterminate or missed interpretations are unavoidable in clinical practice.

In conclusion, our study identified that preoperative imaging features including the presence of EMNs and lymphadenopathy, in combination with several clinicopathologic factors, were found to be significant in predicting IPMN recurrence. Future studies may help determine effective surveillance strategies following the resection of IPMNs.

## Supplementary information

ELECTRONIC SUPPLEMENTARY MATERIAL

## Abbreviations

EMN: Enhancing mural nodule
HGD: High-grade dysplasia
HR: Hazard ratio
ICC: Intraclass correlation coefficient
ICG: International consensus guideline
IPMN: Intraductal papillary mucinous neoplasm
LGD: Low-grade dysplasia
MPD: Main pancreatic duct
PanIN: Pancreatic intraepithelial neoplasia
