# Supplementary material for: Important radiological and clinicopathological risk factors for the recurrence of intraductal papillary mucinous neoplasms after surgical resection
Source: Eur Radiol. 2025 Feb 19;35(8):5004–16. doi: 10.1007/s00330-025-11431-5 (PMC12226678; doi:10.1007/s00330-025-11431-5)

Important radiological and clinicopathological risk factors for the recurrence of intraductal papillary mucinous neoplasms after surgical resection

ELECTRONIC SUPPLEMENTARY MATERIAL

CT acquisition parameters

Due to the retrospective nature of the study, patients underwent CT using various scanner, and acquisition parameters were slightly difference among the scanners. Commonly used scanners used for image acquisition were as follows: SOMATOM Force, SOMATOM Definition Flash, SOMATOM Definition, Sensation 16 (Siemens Healthineers, Erlangen, Germany), IQon – Spectral CT, iCT 256, Ingenuity CT, Brilliance 64 (Philips Healthcare, Amsterdam, the Netherlands), Revolution CT (GE Healthcare, Chicago, IL, USA), and Aquilion ONE (Canon Medical Systems, Otawara, Japan). Commonly used acquisition parameters were as follows: number of channels, 16 – 128; slice thickness, 2 – 3 mm; reconstruction interval, 2 – 2.5 mm; pitch, 0.507 – 1.43; rotation time, 0.33 – 0.75 s; tube voltage, 90 – 120 kVp; tube current, automatic tube current modulation.

Supplementary table 1. Definitions of imaging features

Imaging features	Definitions
**Cyst size**	The longest diameter of "the most suspicious" cyst measured on axial, coronal, or oblique coronal images. If there is no cyst (in main duct type IPMN), cyst size should be recorded as zero
**EMN size**	The longest diameter of the largest EMN measured on axial or coronal/oblique coronal images<br>If there is no EMN, EMN size should be recorded as zero<br>* EMN: any enhancing solid papillary protuberance within the cyst or dilated MPD, regardless of invading adjacent pancreatic parenchyma
**MPD diameter**	The diameter at the maximally dilated portion, measured on axial, coronal, or oblique coronal images
**Thickened/enhancing cyst wall**	Septa or cyst wall thicker than 2 mm in width with contrast enhancement
**Abrupt change in caliber of the pancreatic duct with distal pancreatic atrophy**	Abrupt change in caliber of pancreatic duct and distal pancreatic parenchymal atrophy<br>* Pancreatic parenchymal atrophy: MPD diameter/pancreatic parenchymal width > 0.5
**Lymphadenopathy**	Enlarged peripancreatic lymph node with a short diameter > 10 mm

Note - "The most suspicious" lesion is defined according to the following sequence: 1) cyst with high-risk stigmata, 2) cyst with worrisome feature, and 3) the largest cyst. EMN, enhancing mural nodule; MPD, main pancreatic duct

Supplementary figure 1. The Schoenfeld residual plot for the lymph node metastasis. The plot shows that the risk is higher in the early postoperative period, while the risk gets lower in the late postoperative period. Dashed line indicates approximately 2-year postoperative period, which well separates the period of higher and lower risk of recurrence.

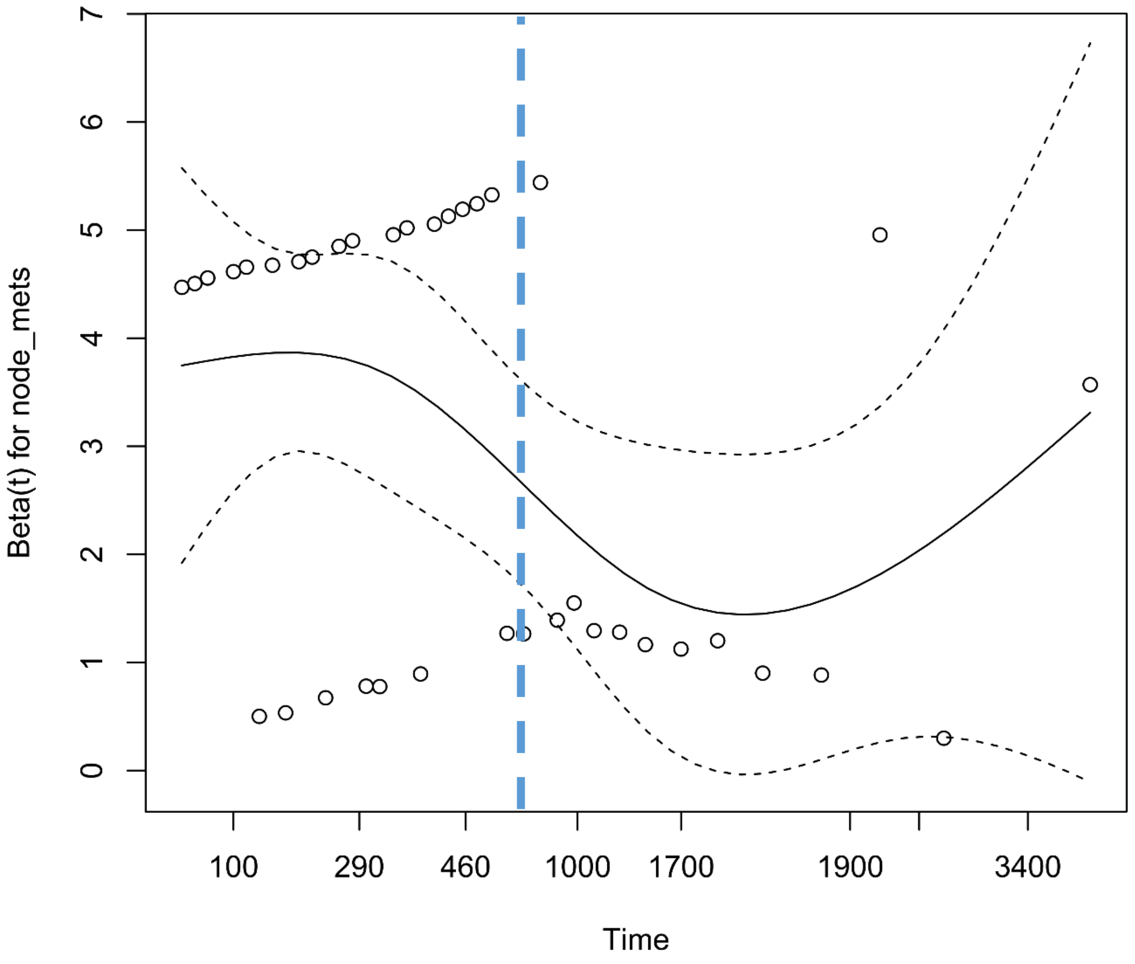

Supplement: Supplementary file 1 — ELECTRONIC SUPPLEMENTARY MATERIAL [file 330_2025_11431_MOESM1_ESM.pdf]
